# HIV screening and linkage to care in a health department in Valencia, Spain: Lessons learned from a healthcare quality improvement project

**DOI:** 10.1097/MD.0000000000030400

**Published:** 2022-10-14

**Authors:** Enrique Ortega-Gonzalez, María Martínez-Roma, María Dolores Ocete, Concepción Jimeno, Antonio Fornos, Amparo Esteban, Magdalena Martinez, Carmen Valero, Neus Gómez-Muñoz, Alba Carrodeguas, Diogo Medina, Miguel Garcia-Deltoro

**Affiliations:** a Fundació Investigació Hospital General Universitari Valencia, Valencia, Spain; b Departament Hospital General València, Valencia, Spain; c Gilead Sciences, Madrid, Spain.

**Keywords:** HIV, late diagnosis, linkage to care, prevention, screening

## Abstract

Spain’s rate of new human immunodeficiency virus (HIV) diagnoses exceeds that of the European Economic Area average (8.6 vs 5.6:100,000 in 2018). The country has failed to meet the first of United Nations Programme on HIV/AIDS (UNAIDS) 90-90-90 targets for HIV control by 2020, with 87.0% of people living with HIV knowing their status, and late presentation rates of 47.6% and 51.5% country-wide and in the Valencian autonomous community, respectively. Advancing screening and linkage to care (SLTC) practices is necessary to effectively control the epidemic. The Valencia Viral Screening (CRIVALVIR) project adopted the TEST model for opportunistic and systematic HIV SLTC in individuals aged 18 to 80 who required blood work for any purpose, as of February 2019. SLTC was integrated into routine clinical workflow across primary care centers serving a population of 360,000 people in Valencia, Spain. Our project successfully upscaled total HIV testing by 194% to over 32,000 patients tested in 14 months. We found an overall prevalence of 0.13% (0.08–0.21) among those screened per protocol (n = 13,061), with foreign-born citizens presenting a 12.5 times significantly higher likelihood of acquiring HIV (95% confidence interval 4.63–33.96, *P* < .0001). We improved late presentation by 18.2 percentage points and prevented an estimated 58 to 70 new secondary infections. HIV screening of the general population in primary care is an effective strategy for achieving timely diagnosis and preventing new infections. Opportunistic, systematic, opt-out approaches are essential to control the HIV epidemic.

## 1. Introduction

The global incidence rate of human immunodeficiency virus (HIV) in Spain (8.6:100,000 in 2018) remains higher than the average for the European Economic Area (EEA, 5.6:100,000), with a prevalence of 0.3% and more than 3000 new cases diagnosed annually.^[1,2]^ Moreover, an estimated 14% of people living with HIV in the EEA are undiagnosed.^[2]^

Approximately half of the new cases identified are diagnosed late, both in Spain (47.6% in 2018) and in the EEA (49.4%).^[1,2]^ Late diagnosis (LD) is defined as the presence of a CD4 count <350 cells/μL in the first determination after HIV diagnosis, and advanced disease (AD) indicated by a CD4 count <200 cells/μL. LD and AD are associated with higher morbidity and mortality rates, particularly in the first year after HIV diagnosis.^[3]^ This situation is more pronounced in women, who have LD in 21.9% and AD in 35.8% of cases, compared with men, 19.2% of whom present with LD and 26.9% with AD.^[2]^ LD could be explained by biases in the perception of risk by professionals and patients.^[4–6]^

Advanced age and non-European origin have also been associated with LD.^[7–10]^ In 2018, foreigners account for 37.6% of new HIV diagnoses in Spain. Of these, 57.6% were from Latin America, followed by 16.2% from sub-Saharan Africa, 10.6% from Western Europe, 8.6% from Central and Eastern Europe, 4.6% from North Africa, and 2.4% from other regions.^[1]^ Migrants remain at high risk of acquiring HIV after moving to countries with more controlled epidemics. They are sexually active within networks of migrant communities, where the HIV prevalence is higher than in the receiving country.^[11]^ There is also evidence that 79% of new HIV infections among migrant sexual gender and minority men from Latin America and the Caribbean living in Europe were acquired postmigration, with a higher likelihood of LD.^[11–13]^

International guidelines recommend enhancing blood-borne virus (BBV) screening and linkage to care (SLTC) practices.^[14]^ Screening is a cornerstone of secondary prevention that reduces disease at its earliest stage, improving the health of those tested and preventing further transmission.^[15]^

Research has shown the impact of HIV treatment in preventing transmission – “Treatment as Prevention” (TasP) – as further HIV transmissions are not expected when people living with HIV (PLWH) are effectively linked to care.^[16]^ Considering the basic HIV reproductive rate (R_0_), or the number of secondary infections generated by 1 PLWH over their lifetime,^[17]^ each diagnosis *and* effective subsequent linkage to care (LTC) is estimated to prevent 3.38–4.14 secondary infections in countries with epidemics similar to Spain (ie, France, the United Kingdom, and Germany).^[18,19]^

In 2006, the United States Centers for Disease Control and Prevention recommended screening individuals between the ages of 13 and 64 who present at healthcare facilities for any clinical reason.^[20]^ In 2014, the Spanish Ministry of Health recommended screening sexually active individuals between the ages of 20 and 59 who present at primary care facilities, require a blood draw for any clinical reason, and live in Spanish provinces with an HIV incidence in the last 3 years above percentile 75.^[21]^ Specifically, in the context of this study, the province of Valencia is in percentile 77. The number of new HIV diagnoses in the Valencian Community still exceeds 400 cases per year, resulting in an average incidence of 9.31:100,000 between 2016 and 2018, of which 51.5% are LD.^[11]^

A previous study in our setting analyzed the attitudes, training, and knowledge of family doctors regarding HIV, and concluded that only 15.2% of them were familiar with Ministry of Health recommendations for the early diagnosis of HIV infection, even though 90% stated that they almost always requested an HIV test after diagnosing another sexually transmitted infection (STI).^[22]^ Doctors who knew the guidelines and had attended a greater number of training activities demonstrated greater knowledge of HIV, and the most experienced conducted greater numbers of HIV tests. Barriers to the early diagnosis of HIV included lack of training of professionals, persistence of stigma, lack of time, and difficulties addressing intimate issues with patients.

In 2018, the European Centre for Disease Prevention and Control released its first evidence-based guidance on integrated BBV testing with a view to achieving the United Nations epidemic control goals for 2030.^[14,23,24]^ Although United Nations Programme on HIV/AIDS (UNAIDS) 90-90-90 goals for control of the HIV epidemic by 2020 (and 95-95-95 by 2030) are an internationally accepted target, most countries struggle to achieve the desired results. Official estimates of Spain’s Ministry of Health show that the country has attained 97.3% of all diagnosed PLWH receiving sustained antiretroviral therapy, 90.4% of whom have achieved viral suppression.^[25]^ However, the country has fallen short of the first target, as only 87.0% of all PLWH (n = 140,000) know their status.^[25]^ This ratio of 13% undiagnosed patients (n = 18,000) means that strategies for prevention and early diagnosis must be intensified and modified.

The CRIVALVIR project for healthcare quality improvement (Valencia Viral Screening, from the Valencian original *Cribratge València Virus*) aimed to advance practices in HIV, hepatitis B virus (HBV), and hepatitis C virus (HCV) SLTC among patients seeking care in Valencia, Spain.

In this article, we have analyzed the project results among individuals seeking primary care and identified success factors and opportunities to improve SLTC efforts.

## 2. Methods

### 2.1. Setting

The project was carried out at the Valencia General Hospital Health Department (*Departament de salut Hospital General de València*, DHGV). DHGV has a catchment population of 360,000, of whom 51.8% are women, with a life expectancy of 82.3 years and a birth rate per thousand deaths of 870.7. Of these, 215,645 people are between 18 and 69 years old, and 50,000 are foreign-born citizens, mainly of African, Romanian, and Chinese origin.^[26]^ Romania accounts for 8.1% of all migrants, China 2.7%, and the African population 4.2%, with Nigeria, Equatorial Guinea, and Senegal being the most represented.

DHGV comprises a general hospital, 26 primary care centers, 5 sexual and reproductive health units, 3 addictive behavior units, and 3 mental health units. DHGV also includes the Picassent Prison, with a capacity for 2000 people.

### 2.2. Quality improvement framework

**Figure 1.**
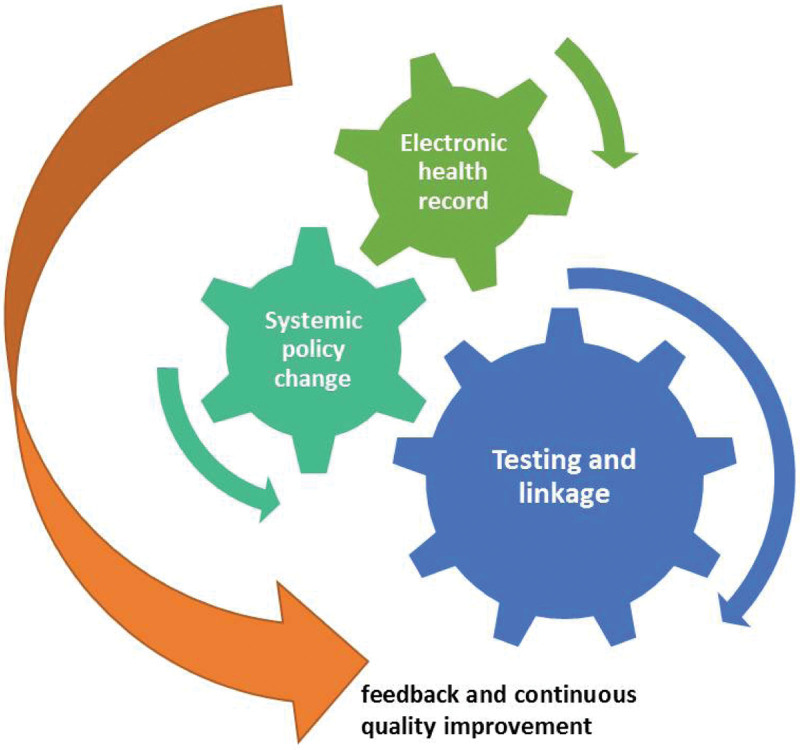
Quality improvement framework of Valencia Viral Screening (*Crivalvir*) project: TEST model of opportunistic and systematic blood-borne virus screening and linkage to care.

The CRIVALVIR project adopted the TEST model of opportunistic and systematic BBV SLTC.^[27]^ TEST consists of 4 guiding components for enhanced SLTC: T, Testing and linkage integrated into the normal clinical flow, using existing clinical infrastructure and staff to create efficiencies; E, Electronic health record (EHR) modification, enhancing efficiencies within EHR and other technologies to facilitate appropriate screening; S, Systemic policy change, implementing institutional and regional policy change to support screening and LTC; and T, Training, feedback and continuous quality improvement, utilizing program data to track progress, identify areas for improvement, and support staff training. Opportunistic screening means integrating screening as part of a clinical encounter for another health condition (eg, adding serologies to specimens processed for other reasons).^[28]^ Systematic screening refers to determining eligibility in all people seeking care, rather than relying on provider or patient initiative. This approach reduces deterring biases while respecting patients’ right to decline, as would occur with other common clinical tests^[27]^ (Fig. 1).

### 2.3. Implementation

We introduced the CRIVALVIR project in February of 2019, screening individuals aged 18 to 80, with a focus on primary care. We hosted BBV knowledge update trainings for primary care doctors and nurses prior to launch. Posters and informative leaflets were prepared and distributed throughout the centers. The screening project was included in management contracts after approval by the Ethics and Research Committee, and written informed consent was required for participation.

Patient pathways were modified to ensure referral to specialist care within 72 hours of a positive diagnosis, while information was shared with the attending family physician through the EHR system. Patients’ attendance at their first appointment with a specialist was followed up and confirmed. In the event of a no-show, the patient or their corresponding social worker were engaged to reattempt LTC.

### 2.4. Diagnostic devices

Serum samples were analyzed using chemiluminescent microparticle immunoassays on the ARCHITECT i2000sr platform. All samples were tested for antibodies against HIV Types 1 and 2 (HIV-1/2) using ARCHITECT HIV Ag/Ab Combo assays. Patient samples with anti-HIV-1/2 antibodies underwent a second test on the same sample with Bio-Rad GeeniusTM HIV-1/2 immunochromatographic assays to confirm and differentiate HIV-1/2 antibodies.

### 2.5. Human rights statements and informed consent

All procedures complied with the responsible committee’s ethical standards on human experimentation and the Helsinki Declaration of 1964 and its later amendments. Informed consent was obtained from all patients included in the project.

## 3. Results

### 3.1. Output indicators

**Figure 2.**
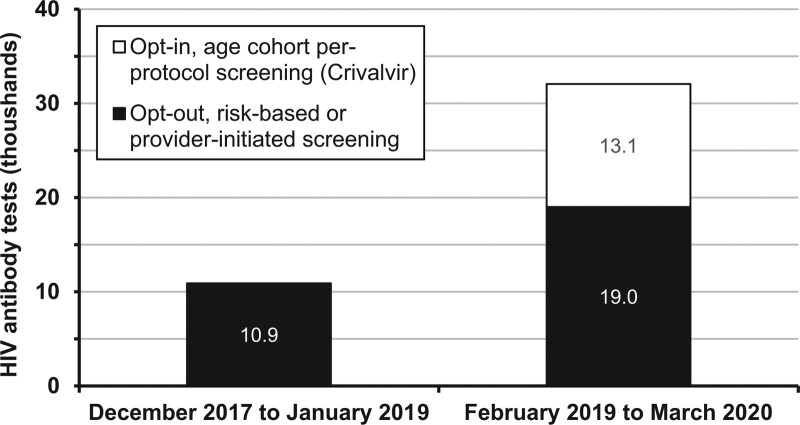
Human immunodeficiency virus screening variation before and after implementation of Valencia Viral Screening (*Crivalvir*). In black: opting out (opt-out).

In the 14 months from February 2019 to March 2020, 32,000 HIV serologies were performed at DHGV, of which 13,061 were performed with written informed consent, as per the CRIVALVIR protocol. The remainder corresponded to risk-based and provider-initiated screening, which represented 74% more tests in this category compared to the same period in previous years (Fig. 2).

**Figure 3.**
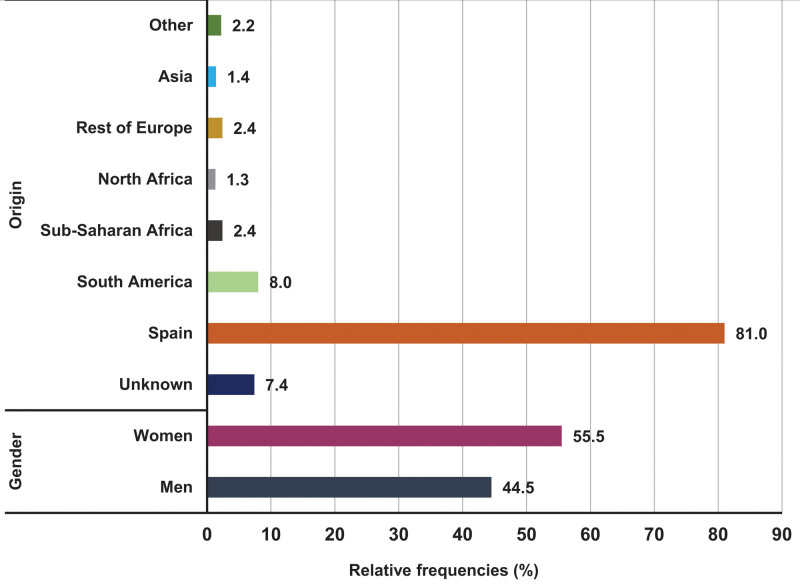
Description of demographic variables of patients tested for human immunodeficiency virus. The results are shown in relative frequencies.

Patients screened per protocol had a mean age of 43.46 ± 13.71 years, 55.5% were women and 44.5% men. In total, 7.4% (n = 962) lacked records on country of birth. In patients for whom nationality data were available, Spaniards accounted for 81%, and the remaining 19% were migrants from South America (8%), sub-Saharan Africa (2.4%), North Africa (1.3%), other European countries (2.4%), Asia (1.4%), and other countries (2.2%) (Fig. 3).

### 3.2. Outcome indicators

HIV was detected in 17 people in the population screened per protocol, with a prevalence of 0.13% [95% confidence interval (CI) 0.08–0.21] and a new infection rate of 5.3:100,000.

The mean age of identified individuals was 40.5 years (SD 13.1), with a higher prevalence in the age ranges of 25–34 (0.15%) and 45–54 (0.20%) years (Table 1).

**Table 1:** Human immunodeficiency virus screening results by age, sex, and nationality.

Age	Population	Screened	HIV positive	%	95% CI	Incidence per 100,000	Prevalence
Total	283,577	13,061	* 17	0.13	0.08–0.21	5.29	5.99
18–25		1141	3	0.26	0.09–0.77		
25–34		2401	4	0.17	0.06–0.43		
35–44		3140	3	0.10	0.03–0.28		
45–54		2922	5	0.17	0.07–0.40		
55–64		2244	1	0.04	0.01–0.25		
65–74		1038	1	0.10	0.02–0.54		
75–80		175	0	0.00	0.00–0.00		
Sex	Population	Screened	HIV positive	%	95% CI	Incidence per 100,000	Prevalence
Male	138,136	5833	10	0.17	0.09–0.32	6.51	7.20
<35		1414	2	0.14	0.04–0.51		
35–54		2818	6	0.21	0.10–0.46		
Female	145,441	7228	7	0.10	0.05–0.20	4.12	4.81
<35		2128	5	0.23	0.10–0.55		
35–54		3244	2	0.06	0.02–0.24		
Nationality	Population	Screened	HIV positive	%	95% CI	Incidence per 100,000	Prevalence
Spain	244,192	10,540	6	0.06	0.03–0.12	1.63	2.45
Other	39,385	1551	11	0.71	0.40–1.27	27.9	27.92

CI = confidence interval, HIV = human immunodeficiency virus.

*Two with previous diagnosis but not linked to care.

We observed a higher prevalence in men (0.17%, n = 10) than in women (0.10%, n = 7), although the difference was not statistically significant [odds ratio (OR) = 1.77, 95% CI 0.67–4.66, *P* = .25), and a new infection rate of 6.5:100,000 for men and 4.1:100,000 for women. In men, the highest prevalence was found in the age group of 35–54 years (0.21%, 0.10–0.46), and in women, below the age of 35 (0.23%, 0.10–0.55). Prevalence was statistically significantly higher in foreign-born citizens (0.71%, n = 11) than in Spaniards (0.06%, n = 6) (OR = 12.54, 95% CI 4.63–33.96, *P* < .0001), resulting in a rate of new infections of 27.9:100,000 for migrants, and 1.6:100,000 for Spaniards.

### 3.3. Impact indicators

All diagnosed patients were linked to care (100%, n = 17), defined as attending a first visit with a specialist postdiagnosis. It is worth noting that 2 of the diagnosed patients (11.8%) were out-of-care, previously known positives, both currently with AD (Table 2).

**Table 2:** Human immunodeficiency virus diagnosed patients by sex, age, nationality, and baseline cluster of differentiation 4 count.

Sex	Age	Nationality	Prior knowledge	Baseline CD4/μl
Male	43	Spain	No	740
Male	44	China (PRC)	No	707
Male	59	Spain	No	525
Male	37	Venezuela	No	476
Male	23	Colombia	No	383
Male	45	Morocco	No	351
Male	31	Nigeria	No	328
Male	65	Spain	No	291
Male	54	Spain	Yes, since 2001	40
Male	47	Bulgaria	No	Unavailable
Female	30	Spain	No	1083
Female	49	Guinea	No	659
Female	52	France	No	260
Female	33	Spain	Yes, since 2002	125
Female	32	Romania	No	53
Female	23	Colombia	No	Unavailable
Female	21	China (PRC)	No	Unavailable

CD4/µL = cluster of differentiation 4 count/microliter, PRC = People’s Republic of China.

The distribution of patients by CD4 level at the time of diagnosis was available in 80% of new cases, with a mean CD4 count of 430 cells/μL. Data show that 8.3% of patients presented with AD and 25.0% with LD, while 66.7% were diagnosed early. LD was found among 20% of the men and 50% of the women, although the difference was not statistically significant.

## 4. Discussion

Implementation of the TEST model in our enhanced SLTC project led to a 3-fold increase of 194% in the number of overall patients screened for HIV at DHGV’s 26 primary care facilities (n = 13,061) when compared to the previous equivalent period, attesting the model’s success in upscaling HIV SLTC outside of the United States.

According to anecdotal data, <10% of patients refused to participate in screening, although no accurate records were kept on such instances.

We found differences in our results compared with those of Spain as a whole. First, the average age (40 years) is slightly higher than that found at the national level (36 years). Even so, we observed 2 age groups with the highest prevalence of new cases: young people under 35 years of age and another 2 decades older, 45–54 years. The male/female sex ratio also varies appreciably in our sample (1:0.69) from the national sample (1:0.18). This could be related to data collection sources, as data are collected mainly in primary care centers, which more often see women.^[29,30]^ This is confirmed by the male/female sex ratio of the total number of people screened in our project (1:1.25). However, the difference in HIV prevalence in women and men was not statistically significant.

Regarding nationality, we observed a high prevalence in foreign-born citizens (0.71%), who had a statistically significant 12.5-fold likelihood of acquiring HIV in comparison with their Spanish peers (0.06%, OR = 12.54, 95% CI 4.63–33.96, *P* < .0001). Migrants represented 64% of positive cases – nearly double the national average of 38%.

Linkage to care (LTC) was remarkably high in our project (100%, n = 17). We attribute this fact to several reasons, including a relatively small number of HIV patients to track, redesign of the patient pathway to ensure LTC in under 72 hours, dedicated headcount to track the completion of LTC and repeat attempts whenever necessary, Spain’s universal healthcare system that ensures care is covered regardless of country of origin or socioeconomic background, and close working relationships between project coordinators and physicians.

Our project helped improve late presentation rates by 18.2 percentage points, which fell from 51.5% in the Valencian Community to 33.3% in our project. We did not observe the longer diagnostic delay in women described by other authors.

Taking into account the estimated R_0_ of the virus in European countries identical to Spain and the fact that 17 cases of HIV-infected people were detected and linked to healthcare, we estimate that 58 to 70 new secondary infections were avoided thanks to the CRIVALVIR project.

Changes in public perception and stigma were apparent in our project: patient adherence to proposed screening was high, and upwards of 32,000 patients were screened in a short time.

Our project also shows an outstanding change in physician awareness regarding the importance of HIV screening, as documented by a 74% increase in risk-based and provider-initiated screening, on top of an already high participation in the per-protocol screening program. We attribute this increase to changes in primary healthcare center management agreements to include productivity in the screening program as an additional indicator of provider performance, the organization of pre- and postlaunch refresher training sessions on BBV at each facility, and continued availability of the project coordinators to respond to physicians’ questions by phone and email.

The spill-over effect from novel opt-in, per-protocol screening (n = 13,061, ie, requiring signed written consent) to opt-out risk-based and provider-initiated screening (n~19,000, +74%, ie, requiring oral consent) can be further explained by physicians’ difficulties in adhering to the written consent procedure. This is due to challenges related to time management, perceived interruption of doctor–patient relationship to explain consent forms during notoriously brief doctor visits, language barriers in explaining consent to migrant patients, logistical challenges involved in collecting and archiving tens of thousands of physical consent forms from over 20 facilities, and the need for case-by-case confirmation of signed consent before inclusion in the per-protocol project pathway. The surprising 45% difference in physician adherence to opt-out compared to opt-in screening found in our project supports the international recommendation that written consent should no longer be considered standard practice for HIV screening, and removing this requirement is effective in increasing testing rates.^[14]^ It may suffice to provide pretest information with materials such as posters or videos in waiting rooms,^[14]^ both of which we used.

It is important to note that we only collected and reported data obtained from patients who provided written consent (n = 13,061). If we were to extrapolate prevalence data from that subset (0.13%, 0.08–0.21) to the remaining group of 19,000 patients screened on the initiative of the provider or due to patient risk factors, we might uncover over 25 more HIV-positive patients, preventing 85 to 104 further secondary infections. It is conceivable that prevalence may have been higher in this unmonitored group due to the use of risk factors, which our protocol does not include as criteria for screening and the likely inclusion of migrants and other patients in whom the consent process may be challenging.

In conclusion, our implementation of the TEST model for BBV SLTC successfully upscaled HIV screening by + 194% to over 32,000 patients, with high patient and provider engagement. We found an overall prevalence of 0.13% among individuals screened per protocol (n = 13,061), with foreign-born citizens presenting a 12.5-fold significantly higher likelihood of acquiring HIV than their Spanish peers. Our project allowed us to improve early diagnosis by 18.2 percentage points and prevent an estimated 58 to 70 new secondary infections.

Screening the general population in primary care centers proved to be an effective strategy for achieving early diagnoses and preventing new HIV infections. Opportunistic, systematic approaches implemented with opt-out consent and primary care health professional training are essential to successfully attain the UNAIDS’ goal of diagnosing 95% of all PLWH.

## Acknowledgments

The authors acknowledge the unique contribution of the hundreds of healthcare professionals of the *Departament Hospital General València* who made this project possible. The authors acknowledge funding from Gilead Sciences’ FOCUS program to support HIV and viral hepatitis screening and linkage to the first medical appointment after diagnosis. FOCUS funding does not support activities beyond the first medical appointment and is agnostic to how partners handle subsequent patient care and treatment. Data collection and management were conducted independently, with additional oversight of an independent data monitoring agency.

## Author contributions

M.M.-R., M.D.O., C.G., A.F., A.E., M.M., C.V., and N.G.-M. supplied and analyzed clinical data from patients. A.C. and D.M. coordinated the study. E.O. and M.G.-D designed and supervised the study. D.M., E.O., and M.G.-D. drafted the manuscript. All authors reviewed and edited the manuscript.
